# Supplementary material for: Prevalence, Risk Factors, and Characterization of Multidrug Resistant and ESBL/AmpC Producing Escherichia coli in Healthy Horses in Quebec, Canada, in 2015–2016
Source: Animals (Basel). 2020 Mar 20;10(3):523. doi: 10.3390/ani10030523 (PMC7143171; doi:10.3390/ani10030523)
Supplement: Supplementary file 1 [file animals-10-00523-s001.zip › FigureS2_questionnaire_english_presentationsurveymonkey.pdf]

Multidrug resistance Horses Owner

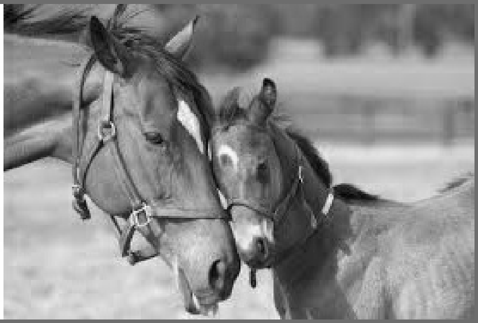

Project presentation : Multidrug resistance in horses in Canada

Context:
Multidrug resistance concerns equine medicine also!
Equine practitioners have to face antimicrobial treatment failure more frequently. This jeopardizes equine health and raises questions concerning environmental contamination and transmission to horse handlers.
Escherichia coli is an intestinal bacterium, mainly harmless, although certain strains may become dangerous. It is widely found in the digestive tracts of all mammals, and is subject to selection pressure during every antimicrobial treatment even if not the targeted bacterial species. It is, therefore, a perfect candidate to become a reservoir of antimicrobial resistance.
One of the main resistance mechanisms of E. coli is β–lactamase production, resulting in the inhibition of an essential antimicrobial class, the β–lactams.
Ceftiofur (Excenel®) is a β–lactam. In Canada, resistance to ceftiofur has developed, due to its extensive use in the meat production industry. In equine medicine, ceftiofur is well tolerated and the new long acting product is very convenient for use in the field. This could facilitate resistance dissemination. Stakeholders in the Equine industry need to maintain the usefulness of this molecule.
Moreover, Ceftiofur resistance is of public health interest as it is responsible for cross resistance to ceftriaxone, the treatment of choice for a certain type of severe, sometimes fatal, diarrhea in children.
The objective of our study is to determine if the normal microflora of horses might represent a reservoir of resistance and play a role in persistence and transmission.
Such data will be useful to elaborate new adapted antimicrobial use guidelines, to improve equine health taking public health into account.

We thank you for your participation to this study. This survey may seem long, however it is very important that you take the time to fill it up with great attention, in order to get data as accurate as possible. Thus we will be able to interpret the results as thouroughly as possible.
If you have any remarks about the questions, or the available answers, please let us know, and we will bring the necessary corrections. Please send an e-mail to maud.de.lagarde@umontreal.ca with the object : survey e coli horse.

Once again : THANK YOU for your time !!!

Multidrug resistance Horses Owner

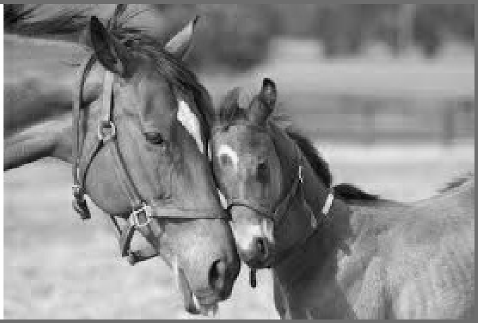

Consent form

Project's tittle : Prevalence of β-lactamase producing, and multi drug -resistant Escherichia coli AND evaluation of risk factors for fecal shedding of these isolates in the equine population in Canada.

Objectives :
Determine if the healthy equine population represents a reservoir of β-lactamase-producing and multidrug resistant E. coli.
Evaluate risk factors associated with fecal shedding of β-lactamase-producing or multidrug-resistant E. coli.
Provide an easily accessible and integrated tool to monitor antimicrobial resistance of clinical E. coli isolates from animals.
Manipulation :
Rectal swab sampling.
Risks and inconveniences for horses :
The sampling represents no risks or inconveniences for the horses.
Time of participation and withdrawal :
My animal's participation to this study is volontary, and I can withdraw him/her anytime without harm.

Confidentiality :
The informations gathered during this study are confidential and will not be used to other ends.
The results will be communicated to the veterinarian who collected the sample, and may be used to be published in a scientific journal.

1. Consent : In the light of the above informations, I agree that my horse/mare participate to the present study.

Multidrug resistance Horses Owner

LABORATOIRE DE RÉFÉRENCE POUR *ESCHERICHIA COLI*

EcL

Université de Montréal

OIE Laboratoire de référence de l'OIE pour *Escherichia coli*

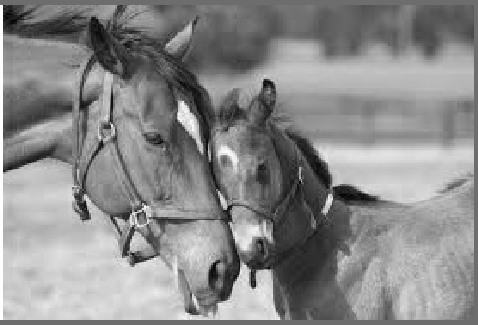

Identification

2. Owner adress

Name

Adress

Adress 2

city

State/Province

Zip code

Country

e-mail Adress

Phone

3. Name of the horse (from now on, we will speak about horse regardless of the sex of your animal).

4. Vet who has realised the sampling

Name

Zip Code

5. Treating vet (if different from the above)

Name [ ]

Zip Code [ ]

Téléphone [ ]

6. Have you been to a vet hospital within the last three months for personal reasons?

- ◯ no, not within the last three months
- ◯ yes, please specify how many times

[ ]

7. Have you been to a human hospital within the last three months for professional reasons?

- ◯ no, not within the last three months
- ◯ yes, please specify how many times

[ ]

8. Have you been to a human hospital for personal reasons, within the last three months

- ◯ no
- ◯ yes, please specify how many times

[ ]

9. Have you been to a human hospital for professional reasons, within the last three months

- ◯ non
- ◯ yes, please specify how many times

[ ]

10. Are you in contact with sick people in your job?

- ◯ no
- ◯ yes

11. Are you in contact with sick animals in your job?

- ○ no
- ○ yes, please specify the animal species

Multidrug resistance Horses Owner

LABORATOIRE DE RÉFÉRENCE POUR *ESCHERICHIA COLI*

EcL

Université de Montréal

OIE Laboratoire de référence de l'OIE pour *Escherichia coli*

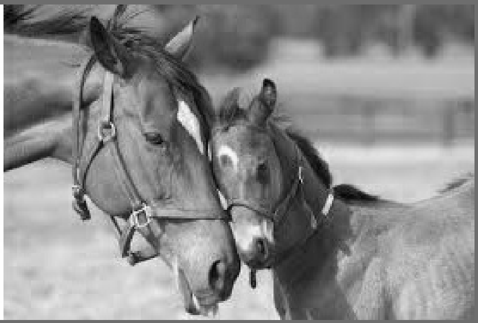

Location

12. Stable adress where your horse stay most of the time

Name

Society

Adress

Adress

City

State/Province

Zip Code

Country

Phone

Multidrug resistance Horses Owner

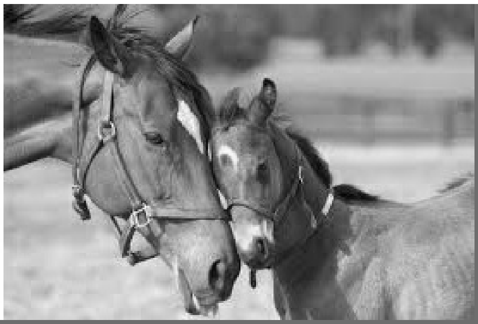

Horse's Signalment

13. For how long have you owned you horse?

☐

14. How old is your horse (in years)

☐

15. What sex is you horse?

- ○ Stallion
- ○ Gelding
- ○ Female
- ○ Female castrated

16. What is your estimation of the weight of your horse

- ○ less than 100kg (200lbs)
- ○ between 100 and 400 kg (201 to 900 lbs)
- ○ between 400 and 600 kg (901 to 1300) lbs
- ○ more than 600 kg (1301lbs)

17. What is the breed of your horse

- ◯ Warmblood
- ◯ Quarter Horse and other
- ◯ Race (Standardbred or Thoroughbred)
- ◯ Draft Horse (Belgium, Clydesdale, Percheron)
- ◯ Canadian, Frisian
- ◯ Pony (including miniature horses)
- ◯ Cross breed (Appendix, etc...)
- ◯ Other, specify

18. What is the main activity of your horse

- ◯ Classical competition (jumper, dressage,cross-country , endurance (national level and above)
- ◯ Classical competition (jumper, dressage, cross-counrty, endurance (provincial level and below)
- ◯ Western competition (national level and above)
- ◯ Western competition (provincial level and below)
- ◯ Leisure horse
- ◯ School horse
- ◯ Race
- ◯ Carriage
- ◯ Reproduction
- ◯ Not at work
- ◯ Other, specify

19. Did you horse take part to equestrian events within the last three months?

- ◯ no
- ◯ yes
- ◯ I do not know

20. If yes, please specify

	Location	Date	Number of participant	Type of event
Event 1				
Event 2				
Event 3				
Event 4				
Event 5				
Event 6				
Event 7				
Event 8				
Event 9				
Event 10				

If the horse have participated to more than 10 events,within the last three months, please, fill the ten last events,and specify the total number of events here.

Multidrug resistance Horses Owner

LABORATOIRE DE RÉFÉRENCE POUR *ESCHERICHIA COLI*

EcL

Université de Montréal

OIE Laboratoire de référence de l'OIE pour *Escherichia coli*

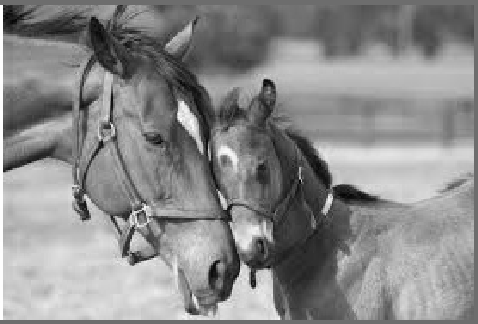

Managment of the horse

If the horse was not sampled in his usual stable, please answer the question concerning the usual stable.

The following questions concerns the most frequent way, at the time of the sampling.

21. Your horse is housed

- ◯ in an individual loose box
- ◯ in a stabling
- ◯ tied in a stall
- ◯ in a pasture
- ◯ Other, specify

22. If your horse has access to a pen, but does not live in a pasture, specify the frequency

- ◯ Once a day
- ◯ Several times a week
- ◯ Once a week and less

23. If your horse has acces to a pen, but does not live in a pasture, specify the other animals he is in contact with in the pen

- ◯ Other horses
- ◯ Alone (my horse is the only one to have access to this pen)
- ◯ Alone (but other horses have access to this pen)
- ◯ With other animals, specify the species

24. Your horse's bedding is

- ◯ Straw
- ◯ Shavings
- ◯ Pasture (no specific bedding)
- ◯ Other, specify

25. Did you use methods or products to control the vermin in your stable, within the last three month?

- no
- yes, we have cats
- yes, specify the type of product, orif you do not know, write "I don't know".

26. Your horse is housed :

- alone
- With other horses (possibility of direct contact)
- With other animals (possibility of direct contact), please specify the species

27. Your horse's housing is cleaned (at least withdrawal of the manure)

- twice a day
- once a day
- several times a week
- once a week
- less than once a week
- my horse is in a pasture that is not cleaned
- other, specify

28. Your horse eats hay

- yes
- no

29. Your horse eats stock feed

- no
- yes, please specify the type, if you know it, otherwise write I do not know

30. Your horse has access to water (indicate the most common water source in each situation, answer only when it is relevant)

	Automatic drinking trough	Bucket	Environmental water source
In his usual housing			
In his pen (if he does not live in his pasture)			

31. Did your horse's feed was modified within the last three months?

- [ ] No
- [ ] Stock feed modification
- [ ] Hay modification
- [ ] Other, please specify the type of modification (i.e :complement addition)

32. How many times does your horse work (here work is defined by either ridden, lunged or harnessed)

- ( ) Does not work
- ( ) less than once a week
- ( ) Once a week
- ( ) Several times a week

33. Did you horse was transported within the last three months

- ( ) yes
- ( ) no
- ( ) I do not know

34. If yes, please specify the location (zip code) where the horse was transported, the date (number of days between transportation and sampling), and the time of transport in minutes

Transport 1 (location zip code)

Transport 1 (number of days between transportation and sampling)

Transport 1 (time of transport in minutes)

Transport 2 (location zip code)

Transport 2 (number of days between transportation and sampling)

Transport 2 (time of transport in minutes)

Transport 3 (location zip code)

Transport 3 (number of days between transportation and sampling)

Transport 3 (time of transport in minutes)

Transport 4 (location zip code)

Transport 4 (number of days between transportation and sampling)

Transport 4 (time of transport in minutes)

Transport 5 (location zip code)

Transport 5 (number of days between transportation and sampling)

Transport 5 (time of transport in minutes)

Transport 6 (location zip code)

Transport 6 (number of days between transportation and sampling)

Transport 6 (time of transport in minutes)

Transport 7 (location zip code)

Transport 7 (number of days between transportation and sampling)

Transport 7 (time of transport in minutes) ____

Transport 8 (location zip code) ____

Transport 8 (number of days between transportation and sampling) ____

Transport 8 (time of transport in minutes) ____

Transport 9 (location zip code) ____

Transport 9 (number of days between transportation and sampling) ____

Transport 9 (time of transport in minutes) ____

Transport 10 (location zip code) ____

Transport 10 (number of days between transportation and sampling) ____

Transport 10 (time of transport in minutes) ____

If your horse was transported more than ten times within the last three months, please give the informations for the last ten trips, and write the number total of trip here : ____

35. How many poeple handle your horse (handle is defined as having direct contact more than once a week)

- ◯ 1 person
- ◯ Between 1 et 5 persons
- ◯ More than 5 persons

36. How many animals arre housed in the same facility (facility is the stable or farm, not the building) that your horse?

	Number
Horses	
Donkeys	
Cows	
Sheeps	
Goats	
Pigs	
Poultry	
Dogs	
Cats	
Camelids	
Other	

37. Have some of these animals been treated with antimicrobials within the last three months?

- ◯ yes
- ◯ no
- ◯ I don't know

38. If you answered yes to the question 37, please specify the treatment if possible

Antimicrobial 1, species and number of treated animal	
Antimicrobial 1, administration route (oral, intramuscular)	
Antimicrobial 1, dosage	
Antimicrobial 1, treatment duration (if known)	
Antimicrobial 2, species and number of treated animal	
Antimicrobial 2, administration route (oral, intramuscular)	
Antimicrobial 2, dosage	
Antimicrobial 2, treatment duration (if known)	
Antimicrobial 3, species and number of treated animal	
Antimicrobial 3, administration route (oral, intramuscular)	
Antimicrobial 3, dosage	
Antimicrobial 3, treatment duration (if known)	
If more than 3 antimicrobials have been used, please specify the rest of the information here	
I do not know	

39. Have your horse been in contact (direct or housed in the same facility) with a hospitalised animal?

- ◯ yes
- ◯ no
- ◯ I do not know

40. If you answered yes tothe question 39, please specify the species

	number	Contact type
Horse		
Donkey		
Cow		
Sheep		
Goat		
Pig		
Poultry		
Dog		
Cat		
Camelids		
I do not know		

Other, please specify the species, the nummber and the contact

Multidrug resistance Horses Owner

LABORATOIRE DE RÉFÉRENCE POUR *ESCHERICHIA COLI*

EcL

Université de Montréal

OIE Laboratoire de référence de l'OIE pour *Escherichia coli*

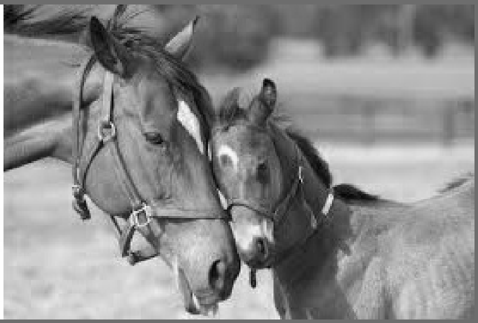

Preventive medicine

The questions concerns the last three months.
Keep going, the questionaire is almost done!!!

41. Have your horse had vet care (other that vaccin, dentale care and deworming) in the last three months?

- yes
- no
- I do not know

42. Is your horse vaccinated against the following?

	Answer	Date
Tetanus		
Rabies		
Herpes (= EHV1 EHV4 = Rhinopneumonia)		
Influenza (= flu)		
West Nile		
Encéphalitidis		
Potomac horse fever		
Strangles		
Botulism		

Other, please specify the vaccine and the date if possible

43. When was your horse dewormed for the last time

- not dewormed
- Less than a month ago
- beween one and three months ago
- more than three months ago
- I do not know

44. If your horse was dewormed, specify the product used the last time if possible

- Exodus Paste
- Panacur
- Pipérazine 34
- Strongid
- Super Pip-Zine
- Worm Clear
- Quest
- Quest plus
- Anthelicide Eq
- I do not know
- Other, please specify

45. Has your horse had medical treatment (not prescribed by a vet), within the las three months?

- yes
- no
- I do not know

46. If you answered yes to question 48, please specify the treatment

Antimicrobial 1 (name molecule or brand name)	
Antimicrobial 1 (Administration route orally intramuscular)	
Antimicrobial 1 (doses)	
Antimicrobial 1 (treatment duration)	
Antimicrobial 2 (name molecule or brand name)	
Antimicrobial 2 (Administration route orally intramuscular)	
Antimicrobial 2 (doses)	
Antimicrobial 2 (treatment duration)	
Other medication 1 (name molecule or brand name)	
Other medication 1 (Administration route orally intramuscular)	
Other medication 1 (doses)	
Other medication 1 (treatment duration)	
Other medication 2 (name molecule or brand name)	
Other medication 2 (Administration route orally intramuscular)	
Other medication 2 (doses)	
Other medication 2 (treatment duration)	
if more medications have been used, please specify here	
I do not know	

47. Did your horse get dental care within the last three months

- ◯ yes
- ◯ no
- ◯ I do not know
