# Supplementary material for: Prevalence, Risk Factors, and Characterization of Multidrug Resistant and ESBL/AmpC Producing Escherichia coli in Healthy Horses in Quebec, Canada, in 2015–2016
Source: Animals (Basel). 2020 Mar 20;10(3):523. doi: 10.3390/ani10030523 (PMC7143171; doi:10.3390/ani10030523)
Supplement: Supplementary file 1 [file animals-10-00523-s001.zip › FigureS1_questionnaire_francais_presentationsurveymonkey.pdf]

Antibiorésistance Cheval Propiétaire

LABORATOIRE DE RÉFÉRENCE POUR *ESCHERICHIA COLI*
EcL
Université de Montréal
OIE Laboratoire de référence de l'OIE pour *Escherichia coli*

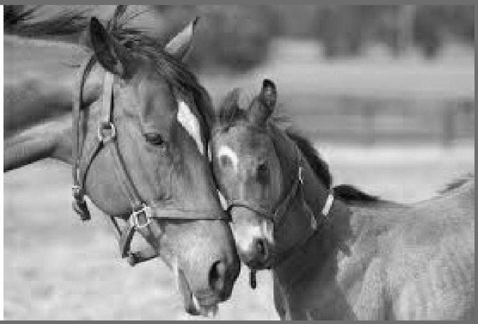

Présentation du projet : Antibiorésistance chez le cheval au Canada

Contexte :
L’antibiorésistance concerne aussi la médecine équine ! Elle pourrait être responsable de la perte d'efficacité de certaines molécules telle que le ceftiofur (Excenel). Les acteurs de la filière équine doivent collaborer, pour perpétuer l’efficacité de cette molécule. D’autant, qu’elle est d’intérêt publique car la résistance au ceftiofur est responsable d’une résistance croisée avec la ceftriaxone, antibiotique de choix dans le traitement des salmonelloses infantile

E. coli subit une pression antimicrobienne à chaque antibiothérapie, ce qui en fait un candidat parfait pour devenir un réservoir de gènes d’antibiorésistance. Un mécanisme de résistance de E. coli est la production de β-lactamases, induisant l'inhibition des β-lactames (pénicillines, céphalosporines).
La flore microbienne intestinale des chevaux représente-t-elle un réservoir de gènes de résistances ? Pour répondre, nous voulons déterminer la prévalence et les facteurs de risque de l'excrétion rectale d'E. coli multirésistant, chez le cheval, au Canada. Ces données permettraient évaluer l'impact de cette espèce dans la propagation de l'antibiorésistance. La connaissance des facteurs de risque associés à l'excrétion rectale est cruciale pour cibler les populations à risque, établir des stratégies pour diminuer la prévalence de l’antibiorésistance et élaborer des recommandations d'utilisation d'antibiotiques.

Nous vous remercions de participer à cette étude. Ce questionnaire peut vous paraître long mais il est très important pour nous que vous preniez le temps de le lire et de le remplir avec attention, de manière à pourvoir interpréter les données le plus précisément possible. Si certaines questions vous semblent confuses ou qu'il manque des possibilités de réponses faites le nous savoir à l'adresse suivante : maud.de.lagarde@umontreal.ca avec comme objet du mail : modifications questionnaire E coli.

Encore une fois MERCI pour votre temps !!!

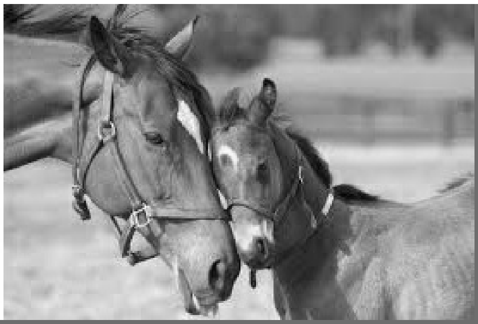

Formulaire de consentement

Titre du projet :

Prévalence et facteurs de risque de l’excrétion fécale de souches E. coli produisant des β-lactamases et de souches multirésistantes dans la population équine canadienne.

Objectifs :
Déterminer si la population équine représente un réservoir de souche de E. coli produisant des β-lactamases et de souches multirésistantes.
Evaluer les facteurs de risque associés à l'excrétion fécale de souche de E. coli produisant des β-lactamases et des souches multirésistantes.

Manipulation :
Prélèvement d'un écouvillon rectal.
Risques et inconvénients :
Le projet ne comporte aucun risque ou inconvénient pour les chevaux.
Durée prévue de la participation volontaire et retrait de la participation :
La participation de mon animal à cette étude est volontaire et je peux le retirer en tout temps sans préjudice.

Confidentialité :
Les informations collectées durant cette étude seront confidentielles et ne seront pas utilisées pour d’autres fins. Ces résultats seront transmis à votre vétérinaire (qui a pris le prélèvement) et pourraient être publiés dans un journal scientifique.

1. Acceptation : À la lumière des informations présentées dans ce document, j’accepte que mon (mes) cheval (chevaux) participent à la présente étude.

LABORATOIRE DE RÉFÉRENCE POUR *ESCHERICHIA COLI*

EcL

Université de Montréal

OIE Laboratoire de référence de l'OIE pour *Escherichia coli*

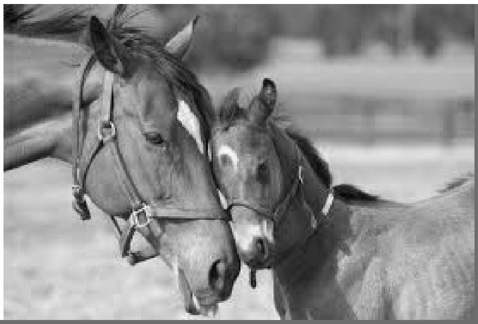

Identification

* 2. Adresse du propriétaire (ces coordonnées restent confidentielles, elles vous sont demandées en cas de besoin pour vous recontacter si certaines réponses demandent des précisions)

Nom

Code postal

Adresse e-mail

Téléphone

3. Nom du cheval :

4. Vétérinaire ayant réalisé le prélèvement ?

Nom

Code postal

5. Coordonnées du vétérinaire traitant (si autre que vétérinaire qui a réalisé le prélèvement)

Nom

Code postal

Téléphone

6. Etes vous allé(e) dans un hôpital vétérinaire dans les trois derniers mois (pour votre cheval ou pour un autre animal) à des fin personnelles?

☐ non

☐ oui, précisez le nombre de fois

7. Etes vous allé(e) dans un hôpital vétérinaire dans les trois derniers mois à des fin professionnelles?

○ non

○ oui, précisez le nombre de fois

8. Etes vous allé(e) dans un hôpital humain dans les trois derniers mois à des fins personnelles?

☐ non

☐ oui, précisez le nombre de fois

9. Etes vous allé(e) dans un hôpital humain dans les trois derniers mois à des fins professionnelles?

○ non

○ oui, précisez le nombre

10. Etes vous en contact avec des personnes malades dans votre métier?

○ non

○ oui

11. Etes vous en contact avec des animaux malades dans votre métier?

○ non

○ oui, précisez l'espèce animale

LABORATOIRE DE RÉFÉRENCE POUR *ESCHERICHIA COLI*

EcL

Université de Montréal

OIE Laboratoire de référence de l'OIE pour *Escherichia coli*

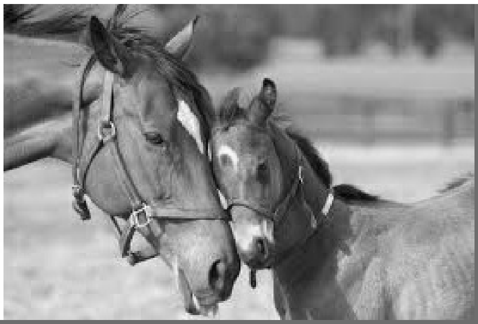

Localisation

12. Adresse de l'écurie où loge votre cheval la majorité du temps

Nom

Adresse

Adresse 2

Ville

État/Province

Code postal

Pays

Téléphone

Antibiorésistance Cheval Propiétaire

LABORATOIRE DE RÉFÉRENCE POUR *ESCHERICHIA COLI*

EcL

Université de Montréal

OIE Laboratoire de référence de l'OIE pour *Escherichia coli*

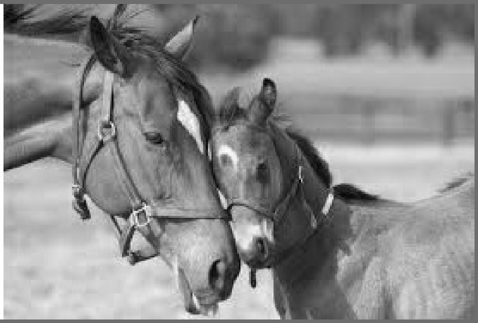

Signalement du cheval

13. Depuis combien de temps avez vous votre cheval?

14. Quel age a votre cheval (ne mettre que le nombre en année)

15. Quel est le sexe de votre cheval?

- Male non castré
- Hongre
- Femelle
- Femelle castrée

16. Vous estimez le poids de votre cheval

- moins de 100kg (200 lbs)
- entre 100 et 400 kg (201 - 900 lbs)
- entre 400 et 600 kg (90-1300 lbs)
- plus de 600 kg (plus de 1301 lbs)

17. Quelle est la race de votre cheval

- Cheval de selle (Warmblood)
- Quarter Horse et famille
- Course (Trotteur ou Thoroughbred)
- Chevaux lourd (Belge, Clydesdale, Percheron)
- Canadien, Frison
- Poney (incluant les chevaux miniature)
- Croisé (Appendix, etc...)
- Autre, Précisez

18. Quelle est l'activité principale de votre cheval (au moment du prélèvement)

- Compétition classique (CSO, dressage, complet, endurance, niveau national et au dessus)
- Compétition classique (CSO, dressage, complet, endurance, niveau provincial et au dessous)
- Compétition western (niveau national et au dessus)
- Compétition western (niveau provincial et en dessous)
- Cheval d'école
- Course
- Attelage
- Reproduction
- Pas au travail
- Autre (veuillez préciser)

19. Votre cheval a t il participé à des événements équestre dans les trois derniers mois?

- non
- oui
- je ne sais pas

20. Si oui, précisez

	Lieu	Date	Nombre de participants	Type d'évènement
Événement 1				
Événement 2				
Événement 3				
Événement 4				
Événement 5				
Événement 6				
Événement 7				
Événement 8				
Événement 9				
Événement 10				

Remarque : Si votre cheval a participé à plus de 10 événements équestres dans les trois derniers mois, remplir les 10 lignes avec les 10 derniers événements et le nombre total d'événements :

Antibiorésistance Cheval Propiétaire

LABORATOIRE DE RÉFÉRENCE POUR ESCHERICHIA COLI

EcL

Université de Montréal

OIE Laboratoire de référence de l'OIE pour Escherichia coli

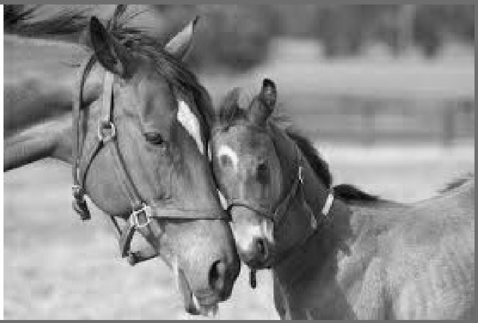

Régie de votre cheval

Remarque : si votre cheval n'est pas prélevé dans son habitât habituel veuillez répondre aux questions sur son habitat habituel.

Les questions de l'hébergement et de l'alimentation concernent la manière la plus fréquente au moment du prélèvement.

21. Votre cheval est hébergé (le plus fréquemment)

- ◯ Dans un box
- ◯ Dans une stabulation libre
- ◯ Attaché dans une stalle
- ◯ Dans un paturâge
- ◯ Autre (veuillez préciser)

22. Si votre cheval a accès à un enclos dehors (mais ne vit pas au pâturage), précisez la fréquence :

- ◯ Une fois par jour
- ◯ Plusieurs fois par semaine
- ◯ Une fois par semaine et moins

23. Si votre cheval a accès à un enclos dehors (mais ne vit pas au pâturage), précisez avec quels autres animaux :

- ◯ Avec d'autres chevaux
- ◯ Seul (mon cheval est le seul à aller dans ce pâturage)
- ◯ Seul (mais d'autres chevaux utilisent ce pâturage)
- ◯ Avec d'autres animaux, précisez l'espèce

24. La litière de votre cheval se compose (le plus souvent) de

- ◯ Paille
- ◯ Copeau
- ◯ Mon cheval vit au pâturage (pas de litière)
- ◯ Autre (veuillez préciser)

25. Avez-vous utilisé des produits ou méthodes de contrôle de la vermine dans votre écurie dans les trois derniers mois?

- ◯ non
- ◯ oui, présence de chats dans l'écurie
- ◯ oui (veuillez préciser le type de produit si possible si vous ne la connaissez pas écrire "je ne sais pas"))

[ ]

26. Votre cheval est hébergé (en contact direct = possibilité de se toucher) le plus souvent (plusieurs réponses possibles):

- ☐ Seul
- ☐ Avec d'autres chevaux
- ☐ Avec d'autres animaux (veuillez préciser l'espèce)

[ ]

27. L'habitat de votre cheval est nettoyé, le plus souvent (nettoyé se définit ici comme au minimum l'élimination des fumiers):

- ◯ deux fois pas jour
- ◯ une fois par jour
- ◯ plusieurs fois par semaine
- ◯ une fois par semaine
- ◯ Moins d'une fois par semaine
- ◯ Mon cheval est au pâturage qui n'est pas nettoyé
- ◯ Autre (veuillez préciser)

[ ]

28. Votre cheval mange du foin

- ◯ oui
- ◯ non

29. Votre cheval mange du grain

- ◯ non
- ◯ oui (veuillez préciser le type de moulée si vous le connaissez si vous ne le connaissez pas écrire "je ne sais pas")

[ ]

30. Votre cheval à accès à l'eau (indiquer la source d'abreuvement la plus courante dans chacune des situations)?

	Par abreuvoir automatique	Par abreuvoir manuel (seaux)	3. Par une source d'eau en contact direct avec l'environnement (exemple : puits, ruisseau, autres)
Dans son habitat le plus habituel			
Dans son enclos (s'il a accès à un enclos mais ne vit pas au pâturage)			

31. Votre cheval a-t-il subit des modifications dans son alimentation dans les trois derniers mois?

- ☐ Non
- ☐ Modification de la moulée
- ☐ Modification du foin
- ☐ Autre (veuillez préciser le type de modification ex : ajout de complémtent)

32. A quelle fréquence votre cheval travaille-t-il (travail se défini comme étant monté, longé ou attelé)?

- ◯ Ne travaille pas
- ◯ Moins d'une fois par semaine
- ◯ Une fois par semaine
- ◯ Plusieurs fois par semaine

33. Votre cheval a-t-il été transporté dans les trois dernier mois?

- ◯ oui
- ◯ non
- ◯ je ne sais pas

34. Si oui, précisez les lieux (code postal) vers où le cheval a été transporté / la date (estimation du nombre de jours écoulé entre le transport et le prélèvement) / la durée de transport (en minutes)

Transport 1 lieu (code postal)

Transport 1 (nombre de jours écoulé entre le transport et le prélèvement)

Transport 1 (en minutes)

Transport 2 lieu (code postal)

Transport 2 (nombre de jours écoulé entre le transport et le prélèvement)

Transport 2 (en minutes)

Transport 3 lieu (code postal)

Transport 3 (nombre de jours écoulé entre le transport et le prélèvement)

Transport 3 (en minutes)

Transport 4 lieu (code postal)

Transport 4 (nombre de jours écoulé entre le transport et le prélèvement)

Transport 4 (en minutes)

Transport 5 lieu (code postal)

Transport 5 (nombre de jours écoulé entre le transport et le prélèvement)

Transport 5 (en minutes)

Transport 6 lieu (code postal)

Transport 6 (nombre de jours écoulé entre le transport et le prélèvement)

Transport 6 (en minutes)

Transport 7 lieu (code postal)

Transport 7 (nombre de jours écoulé entre le transport et le prélèvement)

Transport 7 (en minutes)

Transport 8 lieu (code postal)

Transport 8 (nombre de jours écoulé entre le transport et le prélèvement)

Transport 8 (en minutes)

Transport 9 lieu (code postal)

Transport 9 (nombre de jours écoulé entre le transport et le prélèvement)

Transport 9 (en minutes)

Transport 10 lieu (code postal)

Transport 10 (nombre de jours écoulé entre le transport et le prélèvement)

Transport 10 (en minutes)

Si votre cheval a été transporté plus de 10 fois dans les trois derniers mois, remplir les 10 lignes avec les 10 derniers transport et le nombre total de transport :

35. Combien de personnes manipulent votre cheval en moyenne par jour (manipuler se définit ici comme ayant des contacts directs avec le cheval plus d'une fois par semaine):

- 1 personne
- Entre 1 et 5 personnes
- Plus de 5 personnes

36. Combien d'animaux habitent dans le même établissement (ici établissement se définit comme l'écurie ou la ferme globale et pas simplement le bâtiment) que votre cheval?

	Nombre
Chevaux	
Anes	
Vaches	
Moutons	
Chèvres	
Cochons	
Volailles	
Chiens	
Chats	
Camélidés	

Autre, précisez le nombre et l'espèce

37. Est-ce que certains des animaux sur la ferme ont été traités avec des antibiotiques dans les trois derniers mois

- oui
- non
- je ne sais pas

38. Si vous avez répondu oui à la question 37, veuillez préciser le nombre et le type d'animaux ainsi que le type de traitement si possible

Antibiotique1, espèce et nombre d'animaux traités

Antibiotique1, nom (idéalement molécule, sinon mettre le nom du produit)

Antibiotique1, voie d'administration (orale, intramusculaire)

Antibiotique1, dose

Antibiotique1, durée de traitement (si connu)

Antibiotique2, espèce et nombre d'animaux traités

Antibiotique2, nom (idéalement molécule, sinon mettre le nom du produit)

Antibiotique2, voie d'administration (orale, intramusculaire)

Antibiotique2, dose

Antibiotique2, durée de traitement (si connu)

Antibiotique3, espèce et nombre d'animaux traités

Antibiotique3, nom (idéalement molécule, sinon mettre le nom du produit)

Antibiotique3, voie d'administration (orale, intramusculaire)

Antibiotique3, dose

Antibiotique3, durée de traitement (si connu)

si plus de trois antibiotiques ont été utilisés remplir les informations ici

je ne sais pas

39. Votre cheval a-t-il été en contact avec un/des animal/animaux (cheval ou autres espèce) hospitalisé(s) dans les trois dernier mois?

- oui
- non
- je ne sais pas

40. Si vous avez répondu oui à la question 40, veuillez préciser :

	Nombre	Type de contact
Cheval		
Ane		
Vache		
Mouton		
Chèvre		
Cochon		
Volaille		
Chien		
Chat		
Camélidés		
Je ne sais pas		

Autre (veuillez préciser l'espèce le nombre d'animaux et le type de contact, si connus)

Antibiorésistance Cheval Propiétaire

LABORATOIRE DE RÉFÉRENCE POUR *ESCHERICHIA COLI*

Université de Montréal

Laboratoire de référence de l'OIE pour *Escherichia coli*

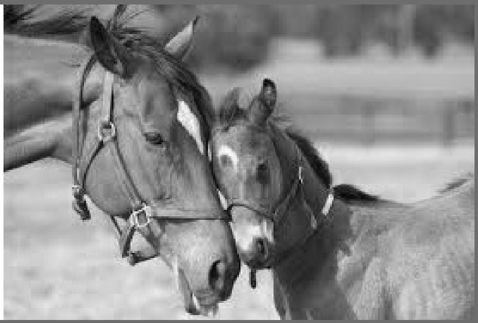

Les informations demandées dans cette page concernent les trois derniers mois précédant le prélèvement.
Courage vous avez bientôt terminé!!!

41. Votre cheval a-t-il été sujet à des soins vétérinaires (autre que vaccins, vermifuge, soins dentaires) dans les trois derniers mois?

- ◯ oui
- ◯ non
- ◯ je ne sais pas

42. Votre cheval est-il vacciné contre les maladie suivantes

	OUI / NON / Je ne sais pas	Date
Tétanos		
Rage		
Herpes (= EHV1 EHV4 = Rhinopneumonie)		
Influenza (= grippe)		
West Nile		
Encéphalites		
Potomac horse fever		
Gourme		
Botulisme		

Autre (veuillez préciser)

43. Quand votre cheval a-t-il été vermifugé pour la dernière fois?

- ◯ Il n'est pas vermifugé
- ◯ Il y a moins de 1 mois
- ◯ Il y a entre un et trois mois
- ◯ Il y a plus de trois mois
- ◯ Je ne sais pas

44. Si votre cheval est vermifugé, précisez avec quel produit il a été vermifugé la dernière fois?

- ◯ Exodus Paste
- ◯ Panacur
- ◯ Pipérazine 34
- ◯ Strongid
- ◯ Super Pip-Zine
- ◯ Worm Clear
- ◯ Quest
- ◯ Quest plus
- ◯ Anthelicide Eq
- ◯ Je ne sais pas
- ◯ Autre (veuillez préciser)

45. Votre cheval a-t-il subit des traitements médicaux<u>non administrés par votre vétérinaire</u> dans les trois mois précédant le prélèvement?

- ◯ oui
- ◯ non
- ◯ je ne sais pas

46. Si vous avez répondu oui à la question 49, veuillez préciser

Antibiotique1 nom (idéalement molécule, sinon mettre le nom du produit)

Antibiotique1 (voie d'administration (orale ou intramusculaire))

Antibiotique1 dose	
Antibiotique1 durée du traitement.	
Antibiotique2 nom (idéalement molécule, sinon mettre le nom du produit)	
Antibiotique2 (voie d'administration (orale ou intramusculaire))	
Antibiotique2 dose	
Antibiotique2 durée du traitement.	
Autre médicament 1 nom (idéalement molécule, sinon mettre le nom du produit)	
Autre médicament 1 (voie d'administration (orale ou intramusculaire))	
Autre médicament 1 dose	
Autre médicament 1 durée du traitement.	
Autre médicament 2 nom (idéalement molécule, sinon mettre le nom du produit)	
Autre médicament 2 (voie d'administration (orale ou intramusculaire))	
Autre médicament 2 dose	
Autre médicament 2 durée du traitement.	
Si d'autres médiaments ont été utilisé, précisez les détails ici	
je ne sais pas	

47. Votre cheval a-t-il reçu des soins dentaires au cours des trois derniers mois?

- ◯ oui
- ◯ non
- ◯ je ne sais pas
